# Intravital imaging reveals spatiotemporal dynamics of oncolytic *Salmonella* YB1-induced intratumoral vascular thrombosis and tumor targeting

**DOI:** 10.3389/fimmu.2025.1733164

**Published:** 2026-01-16

**Authors:** Bin Yu, Lei Shi, Weiwang Duan, Dongmei Cui, Edwin R. Manuel, Wei Huang

**Affiliations:** 1 Department of Research and Development (R&D Department), Shanghai Salvectors Biotech Ltd, Shanghai, China; 2 School of Biomedical Sciences, The University of Hong Kong, Hong Kong, Hong Kong SAR, China; 3 Department of Gastrointestinal Medical Oncology, The University of Texas MD Anderson Cancer Center, Houston, TX, United States; 4 Department of Immuno-Oncology, Beckman Research Institute of the City of Hope, Duarte, CA, United States; 5 State Key Laboratory of Quantitative Synthetic Biology, Shenzhen Institute of Synthetic Biology, Shenzhen Institutes of Advanced Technology, Chinese Academy of Sciences, Shenzhen, China

**Keywords:** cancer therapy, intravital imaging, *Salmonella* YB1, thrombosis, tumor vasculature

## Abstract

**Introduction:**

In recent years, oncolytic bacterial therapy has emerged as a promising strategy in cancer research due to its unique advantages in tumor targeting and immune activation. Among various bacterial candidates, *Salmonella* demonstrates exceptional potential owing to its amenability to genetic engineering and its capacity to serve as an efficient vector for therapeutic gene delivery. However, the precise spatiotemporal dynamics of the interaction between *Salmonella* and tumor vasculature, as well as the mechanisms by which *Salmonella* targets and colonizes tumors via the circulatory system, remain to be fully elucidated.

**Methods:**

A dorsal skin-fold window chamber model was established in nude mice bearing tdTomato-labeled MDA-MB-231 xenografts. Real-time intravital imaging was used to track tumor growth, angiogenesis, and EGFP-labeled YB1 distribution after intravenous administration.

**Results:**

Following intravenous injection, YB1 was retained in local vascular regions within the characteristically disordered tumor vascular network, such as "Shoulder Structure" or "Maze Structure". This physical entrapment facilitated direct interaction between YB1 and vascular endothelial cells, leading to endothelial damage and subsequent intratumoral vascular thrombosis. This process effectively blocked the tumor's blood supply and induced local hypoxia. Importantly, the formation of thrombosis and the hypoxic microenvironment further promoted the colonization and proliferation of YB1 within the tumor parenchyma, ultimately achieving effective tumor targeting and regression.

**Discussion:**

This study reveals the novel mechanism of YB1's tumor targeting and colonization from the perspective of interaction with tumor vasculature. These findings providing critical theoretical support for the future design of more efficient and safer oncolytic bacterial therapies and lay a foundation for YB1’s clinical optimization.

## 1 Introduction

Solid tumors represent a major global health threat (1), posing significant therapeutic challenges due to their invasive growth, high metastatic potential, and resistance to conventional treatments such as surgery, radiotherapy, and chemotherapy (2). Despite notable advances in targeted (3) and immunotherapies (4), many cancers exhibit high recurrence rates and poor prognoses, highlighting the urgent need for innovative therapeutic strategies. Oncolytic bacterial therapy (5–7) has emerged as a highly promising novel anticancer approach, owing to its inherent tumor-targeting ability, selective proliferation within the unique tumor microenvironment, and potential to induce local anti-tumor immune responses. As early as the late 19th century, William Coley (8) achieved remarkable success by treating sarcoma patients with Coley’s toxins. In recent years, with the rapid development of molecular and synthetic biology, an increasing number of bacteria (9–12) have been engineered for cancer therapy. For instance, the attenuated *Salmonella typhimurium* VNP20009 (13, 14) (with deletions in *msbB* and *purI* genes) and the leucine-arginine auxotrophic *S. typhimurium* A1-R (15) have both demonstrated significant anti-tumor activity in various preclinical tumor models. Our team’s previously constructed hypoxia-responsive *Salmonella* YB1 (16) has also been shown to effectively inhibit the growth of multiple tumors (17–20), including breast, colon, and liver cancers, as well as neuroblastoma. A key phenomenon revealed by these studies is that regardless of the genetic engineering strategy employed, *Salmonella* exhibits a remarkable tropism for tumors, reaching enrichment ratios of up to 1000:1 in tumors versus normal organs. This suggests the existence of critical structural differences between tumor and normal tissues. Extensive research has confirmed that the vasculature of solid tumors displays significant pathological features (21, 22), including abnormal angiogenesis, a disorganized network structure, dysfunction, increased permeability, and sluggish blood flow. These characteristics not only drive tumor growth and metastasis but also severely impede the effective delivery of conventional anticancer drugs. Consequently, targeting tumor-associated vasculature (23) is considered a highly attractive strategy in cancer therapy, aiming to inhibit tumor growth by cutting off its nutrient and oxygen supply.

Early studies (24, 25), used intravital microscopy to directly observe the localization and behavior of *Salmonella* within solid tumors. However, their research also noted that bacteria struggled to adhere firmly to high-velocity vessels. Although interactions occurred at low flow rates, ultimately only a very small fraction (approximately 0.035% ± 0.015%) of bacteria achieved stable adhesion in tumor vessels. The mechanism of this adhesion and the ultimate fate and functional impact of these attached bacteria remain unclear. Furthermore, Leschner et al. (26) reported that tumor-colonizing bacteria cause hemorrhage and induce widespread vascular disruption within 1–3 days by rapidly activating pro-inflammatory cytokines such as TNF-α, IL-6, and MCP-1. These findings provide clues that bacteria may induce vascular damage during the early stages of tumor colonization. Nevertheless, the precise spatiotemporal dynamics of the interaction between bacteria and the tumor vasculature, and how they specifically affect key vascular systems within the tumor to ultimately cause its regression, require further elucidation.

To address these critical scientific questions, this study combines intravital imaging technology with a dorsal skin-fold window chamber model (27) in tumor-bearing mice. We first observed the real-time processes of solid tumor growth, angiogenesis, and the formation of hypoxic regions. Subsequently, we conducted a detailed investigation into the interactions among bacteria, tumors, and the tumor vasculature, as well as the mechanisms of YB1’s tumor targeting and anti-tumor effects. This work aims to lay a solid foundation for the further optimization of oncolytic bacteria for clinical applications in cancer therapy.

## 2 Results

### 2.1 Establishment of the animal model and intravital imaging system

**Figure 1:**
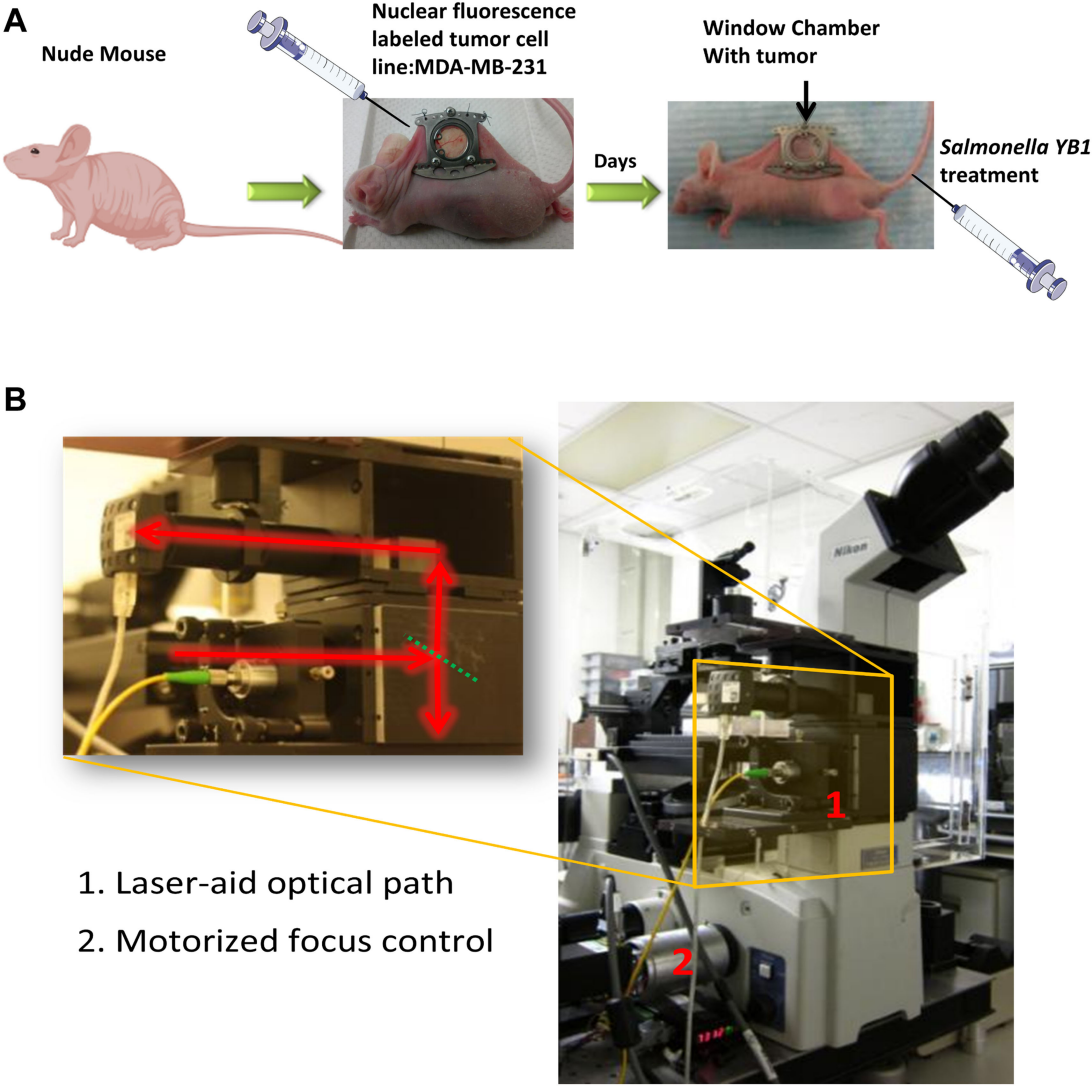
Animal model and intravital imaging system. **(A)** The ‘Window Chamber’ intravital imaging system. The process of *salmonella* treatment is traced using the ‘Window Chamber’ model. Nude mice were implanted with nuclear fluorescence-labeled cancer cells. **(B)** Autofocusing imaging microscope system.

To achieve a comprehensive and dynamic observation of tumor-bacteria-vasculature interactions, we combined imaging technology with a dorsal skin-fold window chamber model (**Figure 1A**). This integrated system enabled real-time *in vivo* visualization of tumor vascular responses during bacterial therapy. This sophisticated model supports the real-time observation of dynamic changes within mouse tissues, allowing for continuous tracking of tumor progression, therapeutic effects, and angiogenesis for 2 to 3 weeks post-implantation. All intravital observations were performed using a Nikon inverted microscope (Eclipse Ti-s/L 100; **Figure 1B**). Through this integrated imaging system, we successfully tracked the *in vivo* proliferation of tumor cells and the dynamic movement of bacteria.

**Figure 2:**
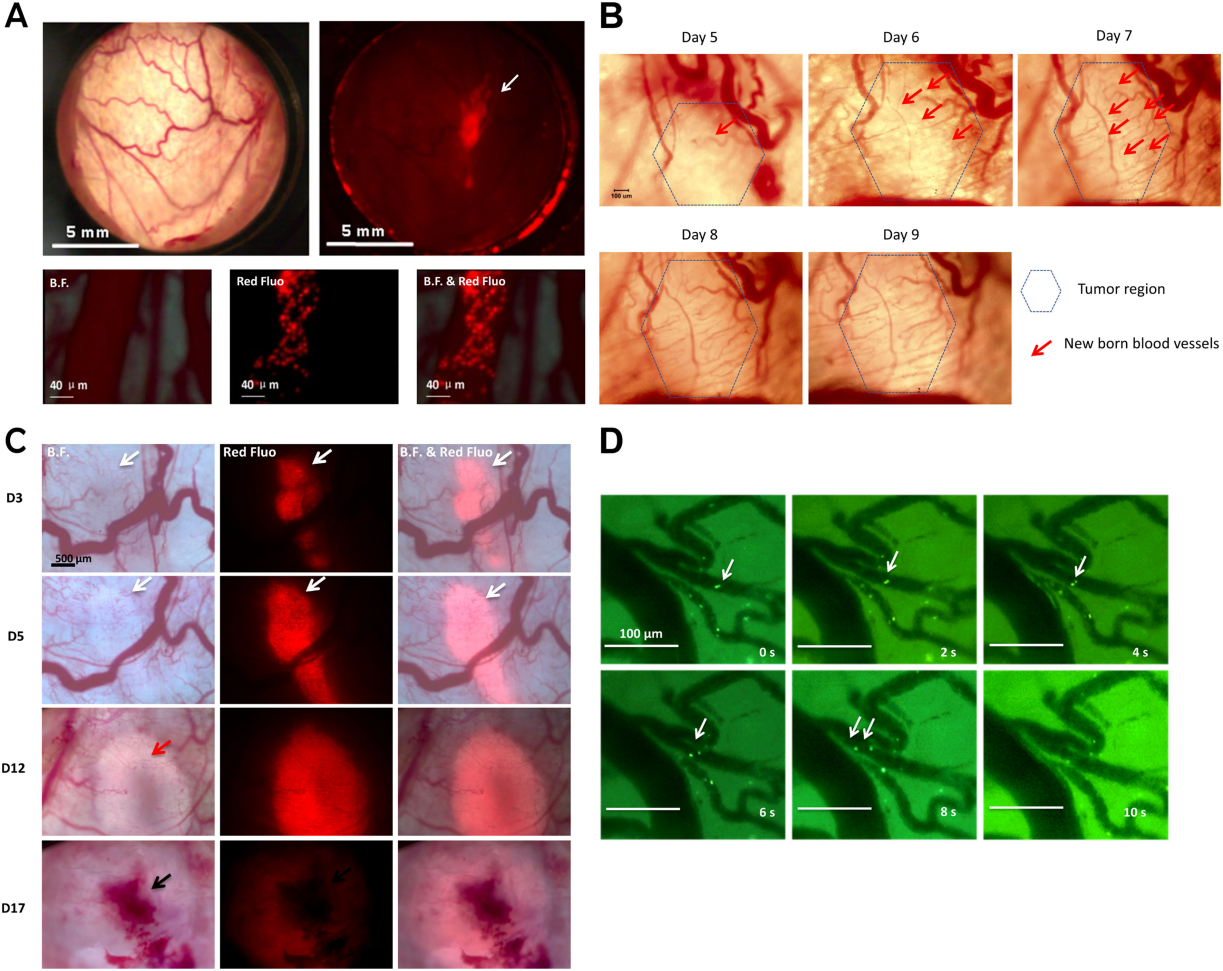
*In vivo* real-time imaging of tumor growth, angiogenesis, and *salmonella* distribution in the window chamber model. **(A)** Transmission bright field imaging of blood vessels and fluorescence imaging of tumors three days post-implantation. Blood vessels and tumor cells were observed growing around the vessels in both bright and fluorescent fields. White arrow indicate tdTomato-labeled MDA-MB-231 cancer cells. **(B)** Images captured under the ‘Window Chamber’ model. **(C)** Observation of tumor formation in the Window Chamber model. The tumor growth status of the same mouse was tracked from Day 3 to Day 17. Red signal: tdTomato-labeled MDA-MB-231 cancer cells. White arrow: tumor cells; Red arrow: tumor-induced angiogenesis; Black arrow: hypoxic region. **(D)** Time-lapse tracking of *Salmonella in vivo*. Tracking a single YB1 cell inside vessels. Two YB1 cells were moving from a small vessel to a larger one, with each panel captured after 2 seconds. White arrow: YB1.

To facilitate direct observation of tumor cells, we stably transfected the MDA-MB-231 tumor cell line with tdTomato, a red fluorescent protein. Continuous monitoring using the dorsal skin-fold window chamber allowed us to simultaneously track tumor progression, angiogenesis, and hypoxia (**Figure 2A**). Initial tumor formation/growth was detected on day 3 post-implantation. Between days 5 and 9 post-implantation, small vascular sprouts and extensions growing from existing vessels were observed at the periphery of the cell clusters (**Figure 2B**), indicating early angiogenesis. Two weeks post-implantation, the area of the necrotic/hypoxic core at the tumor center significantly increased (**Figure 2C**), primarily due to the inability of tumor cells to obtain sufficient nutrients and oxygen from a sustained lack of blood supply. Subsequently, we assessed the feasibility of using optical imaging to observe the *in vivo* migration and colonization of YB1. To this end, EGFP-labeled YB1 was injected via the tail vein into healthy mice, and its migration within the skin vasculature was tracked using the green fluorescent signal (**Figure 2D**). In summary, our intravital imaging approach successfully enabled the observation of MDA-MB-231 tumors using red fluorescence microscopy, the movement of YB1 in blood vessels using green fluorescence microscopy, and tumor angiogenesis using bright-field microscopy.

### 2.2 YB1 induces destruction of tumor-associated vascular structures *in vitro* and *in vivo*

**Figure 3:**
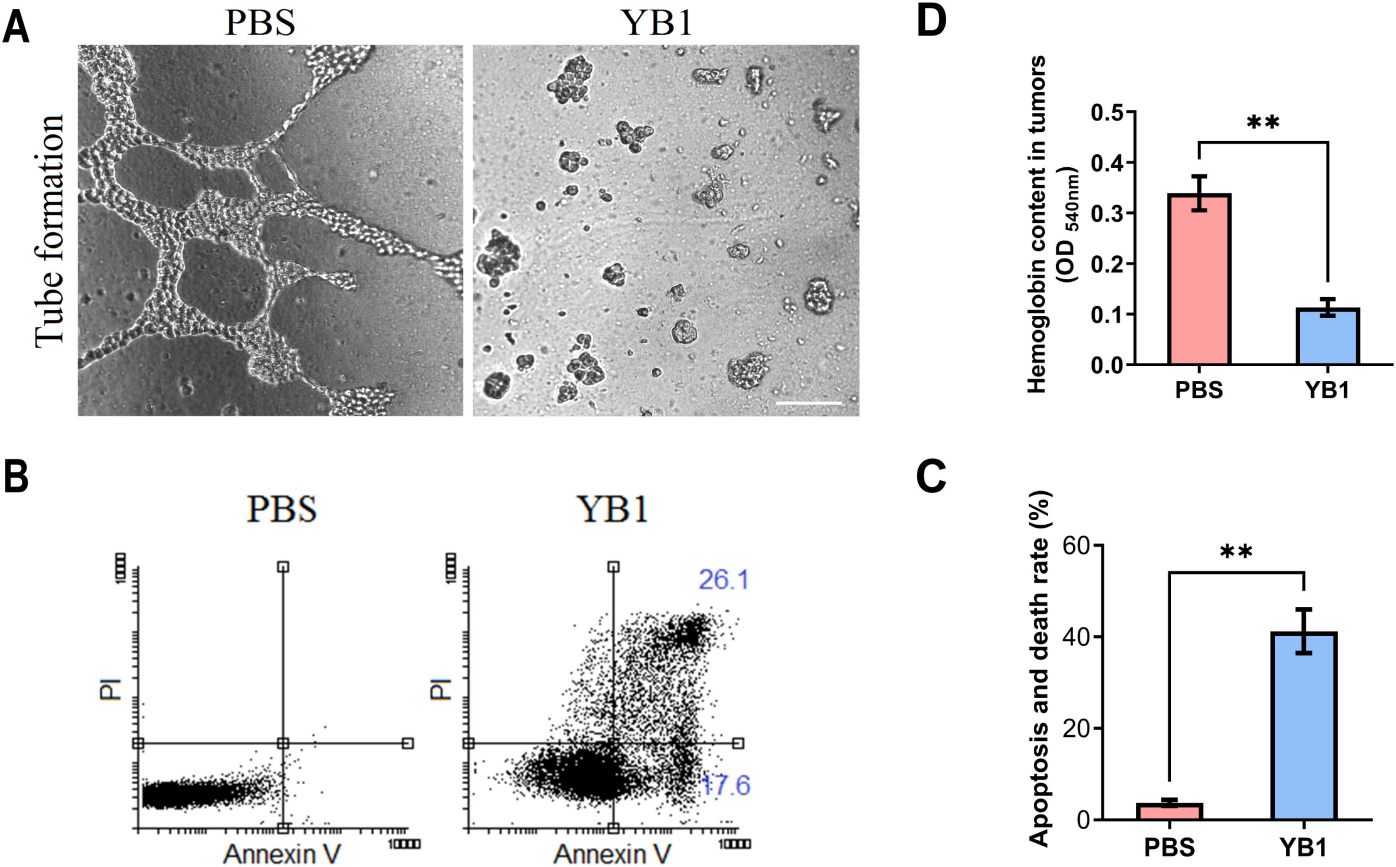
*In vitro* and *in vivo* effects of YB1 on endothelial cells. **(A)** Tube formation in HUVEC culture after incubation with YB1. Images taken at 100x magnification, phase contrast. **(B)** Flow cytometry analysis of cell apoptosis. Q1: Annexin V^-^/PI^+^, cell debris or mechanically damaged cells; Q2: Annexin V^-^/PI^-^, live cell; Q3: Annexin V^+^/PI^-^, early apoptosis; Q4: Annexin V^+^/PI^+^, late apoptosis/necrosis. **(C)** Flow cytometry analysis of apoptosis and death rate. **(D)** Hemoglobin content in tumors after YB1 treatment. **p < 0.01. Data are presented as mean ± SEM (n = 3 per group).

To elucidate the mechanisms of YB1-mediated vascular interaction, we investigated the potential effects of YB1 on endothelial cell integrity and apoptosis *in vitro*. Our findings indicate that YB1 can directly interact with vascular endothelial cells, causing endothelial cell damage and subsequent apoptosis, thereby disrupting vascular structures. Given that angiogenesis relies primarily on the endothelial cell tube formation (28), we evaluated the effect of YB1 on this process. Human Umbilical Vein Endothelial Cells (HUVECs, 4×10^4^ cells/mL) were treated with YB1 (4×10^5^ cfu/mL) at a multiplicity of infection (MOI) of 10 for 6 hours. As shown in **Figure 3A**, YB1 treatment completely inhibited the tube formation. Furthermore, we quantified the apoptotic HUVEC population by flow cytometry (**Figures 3B, C**), which revealed that approximately 40% (The sum of Annexin V^+^/PI^-^ early apoptosis and Annexin V^+^/PI^+^ late apoptosis/necrosis) of endothelial cells underwent apoptosis upon infection with YB1 at an MOI of 10. These *in vitro* results strongly suggest that YB1 can directly damage endothelial cells, inhibit tube formation, providing a solid theoretical basis for further investigation of its tumor vascular destruction effects in animal models.

To assess the tumor vascular-disrupting capability of YB1 *in vivo*, we administered YB1 to athymic nude mice bearing MDA-MB-231 xenografts. Six days post-treatment, tumors were excised, and their hemoglobin concentration was measured as a quantitative indicator of vascular density. The results showed that the tumor hemoglobin levels in the YB1-treated group were significantly lower than those in the control group (*p* < 0.01) (**Figure 3D**), indicating a significant decrease in tumor vascular density. Based on these findings, we confirmed that YB1 can induce the destruction of tumor-associated vascular structures both *in vitro* and *in vivo*.

### 2.3 Early-phase dynamics of YB1-induced tumor vascular disruption and intratumoral colonization

Previous studies (26, 29) have shown that the abnormal structure of tumor vasculature, characterized by increased permeability and sluggish blood flow, provides bacteria with more opportunities and longer interaction times with the vessel wall—a pathological environment crucial for *Salmonella* colonization. We propose two primary mechanisms for YB1-induced tumor vascular disruption: (1) Bacteria become directly entrapped within the rough and irregular lumen of tumor-associated vessels (“Shoulder Structure”) and cause local coagulation by repeatedly impinging on nearby endothelial cells through active swimming. (2) Bacteria enter the complex tumor vasculature (“Maze Structure”) and cause local coagulation by repeatedly impacting the endothelial cells of nearby structures through active swimming.

**Figure 4:**
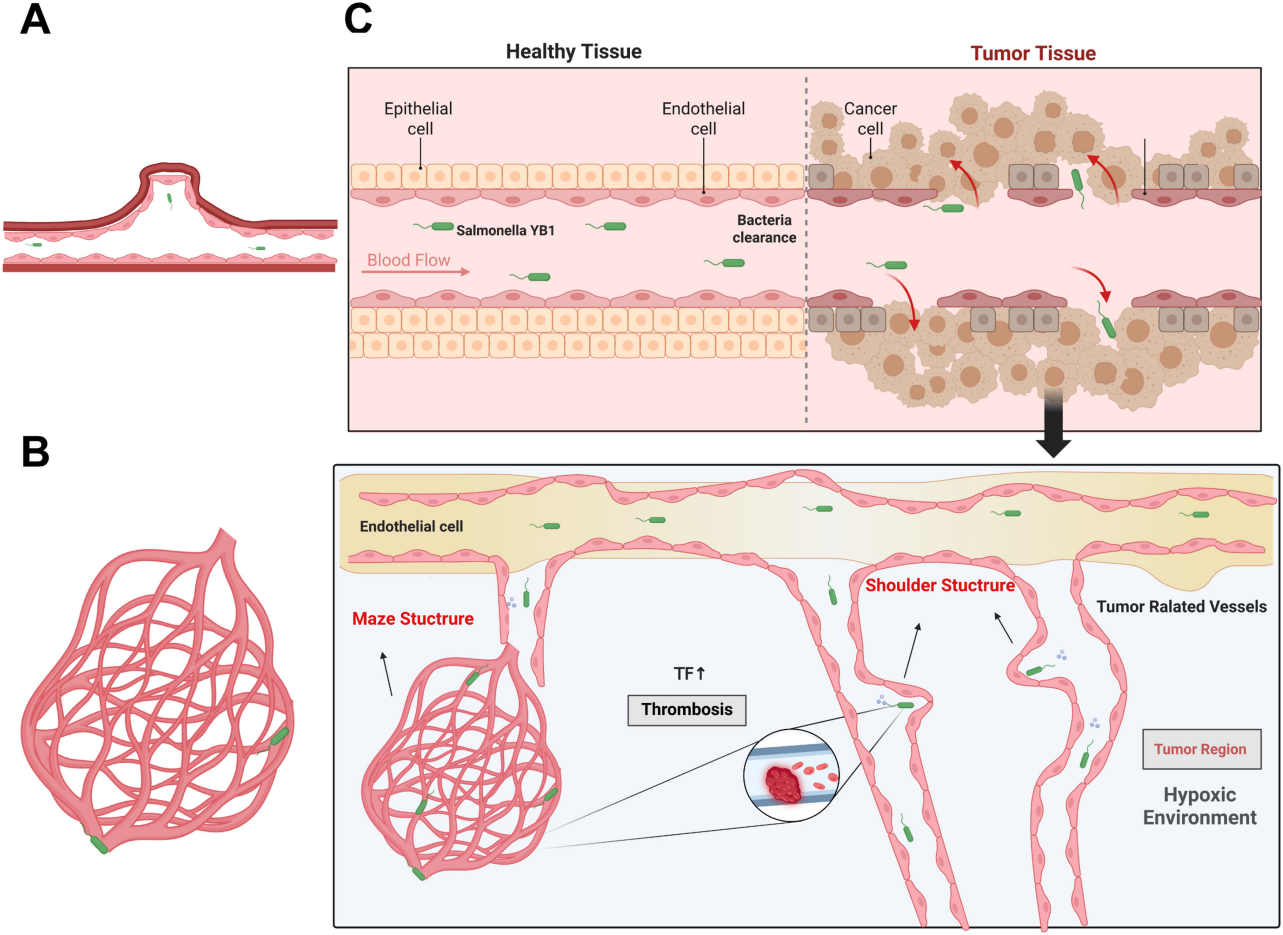
Mechanism diagram of YB1-induced intratumoral vascular thrombosis and intratumoral colonization. **(A)** Shoulder Structure. **(B)** Maze Structure. **(C)** Mechanism Diagram of YB1-Induced Intratumoral Vascular Thrombosis and Intratumoral Colonization.

**Figure 5:**
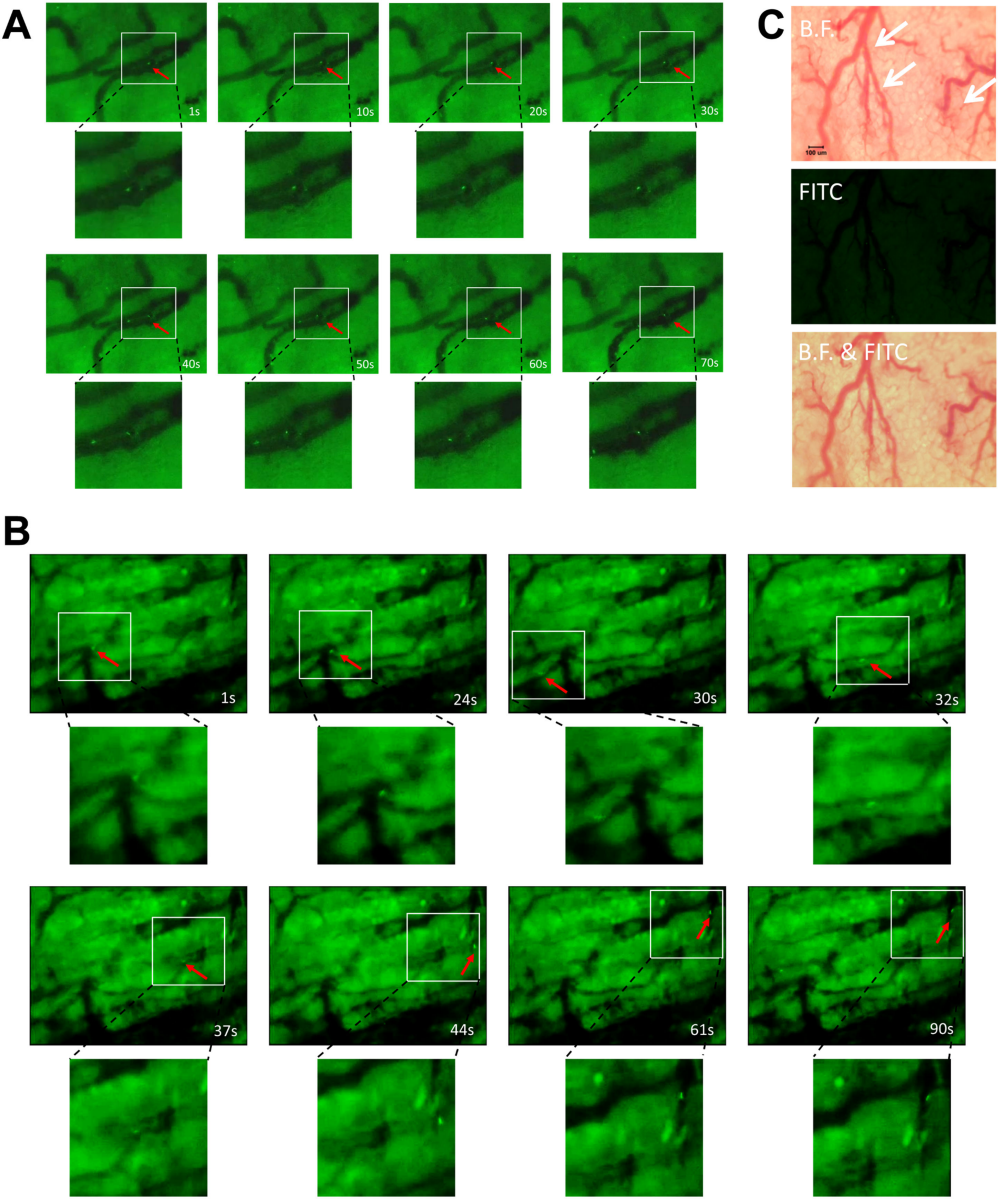
Time-lapse tracking of *Salmonella* in tumor related vessel. **(A)** Time-lapse tracking of *Salmonella* in tumor related vessel (shoulder structure). Red arrow: YB1. **(B)** Time-lapse tracking of *Salmonella* in tumor related vessel (maze structure). Red arrow: YB1. **(C)** Images of normal vessels and *Salmonella* within 30mins. White arrow: normal vessels. **Figure 5A** and **Figure 5B** lack scale bars and these images are primarily intended to illustrate dynamic processes rather than precise dimensional measurements.

To systematically elucidate the dynamic process of YB1’s interaction with and disruption of tumor vasculature, our intravital imaging studies using EGFP-labeled YB1 confirmed and detailed the interplay between YB1 and tumor vascular structures and the progression of bacterial colonization. Following the intravenous injection of 5×10^7^ cfu of EGFP-YB1 into tumor-bearing mice, the bacteria were rapidly distributed throughout the systemic circulation. Within the first 30 minutes, YB1 was detected flowing rapidly in most tumor-associated vessels. However, a portion of the bacteria was observed to be specifically retained in local vascular regions with morphological features resembling “Shoulder Structure” (**Figures 4A, C**, **5A**; **Supplementary Figure 1**; **Supplementary Videos 1**, **2**, **5**). Within these areas of irregular endothelial protrusions, bacteria continuously interacted with and mediated damage to endothelial cells, and some tumor-associated vessels exhibited evident thrombosis leading to vascular occlusion. In addition to “Shoulder Structure,” some tortuous and disordered complex tumor vessels possessed “Maze Structure” morphological features (**Figures 4B, C**, **5B**; **Supplementary Video 3**, **6**). This led to the physical entrapment of YB1 upon entry, resulting in sustained mechanical impact on nearby vascular endothelial cells, which ultimately triggered local coagulation and thrombus formation. Importantly, this cessation of blood flow occurred specifically in tumor-associated vessels, while normal vessels remained unaffected (**Figure 5C**, **Supplementary Video 4**).

### 2.4 Late-phase dynamics of YB1-induced tumor vascular disruption and intratumoral colonization

**Figure 6:**
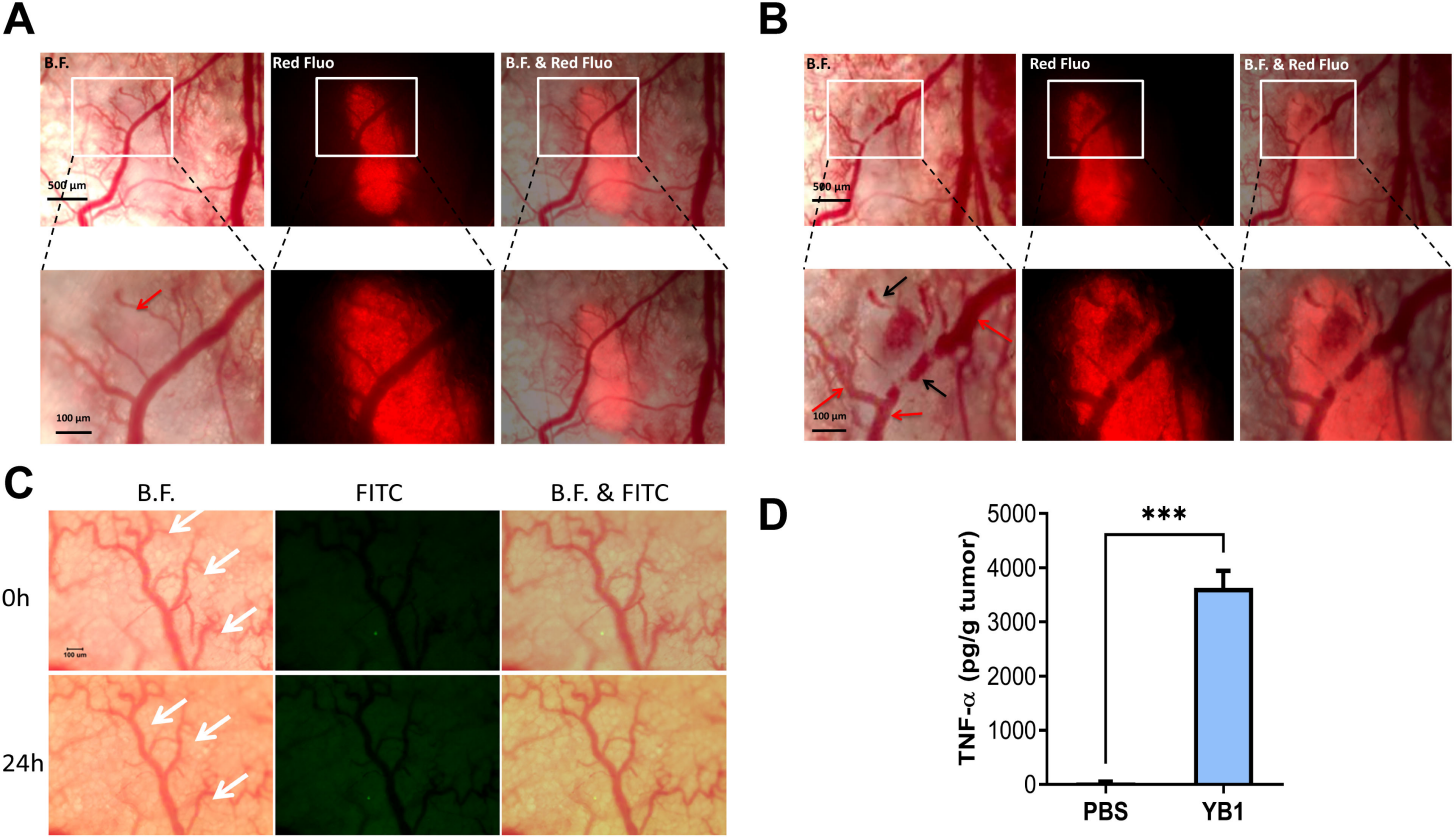
Late-phase of YB1-induced tumor vascular disruption. **(A)** Within 120 minutes after treatment, circulation in the tumor appeared healthy. **(B)** After 24 hours, vessels within the tumor were damaged. Red signal: tdTomato-labeled cancer cells. Red arrows: functional blood vessels; Black arrows: damaged blood vessels. **(C)** Images of normal vessels and *Salmonella* after 24 hours. White arrow: normal vessels. **(D)** ELISA assay results for TNF-α levels. ***p < 0.001. Data are presented as mean ± SEM (n = 3 per group).

Between 30 minutes and 24 hours post-treatment, YB1 persisted in the tumor microenvironment, either trapped within damaged tumor vessels or infiltrating the surrounding tumor tissue. Concurrently, circulating free YB1 in the bloodstream was efficiently cleared by the activated host innate immune system, accompanied by an increase in the concentration of tumor necrosis factor-alpha (TNF-α) in the tumor (**Figure 6D**). Although some tumor-associated vessels did not show immediate damage within 30 minutes (**Figure 6A**), significant damage and occlusion were typically observed within 24 hours post-treatment (**Figure 6B**). In contrast, the normal vasculature remained intact (**Figure 6C**).

**Figure 7:**
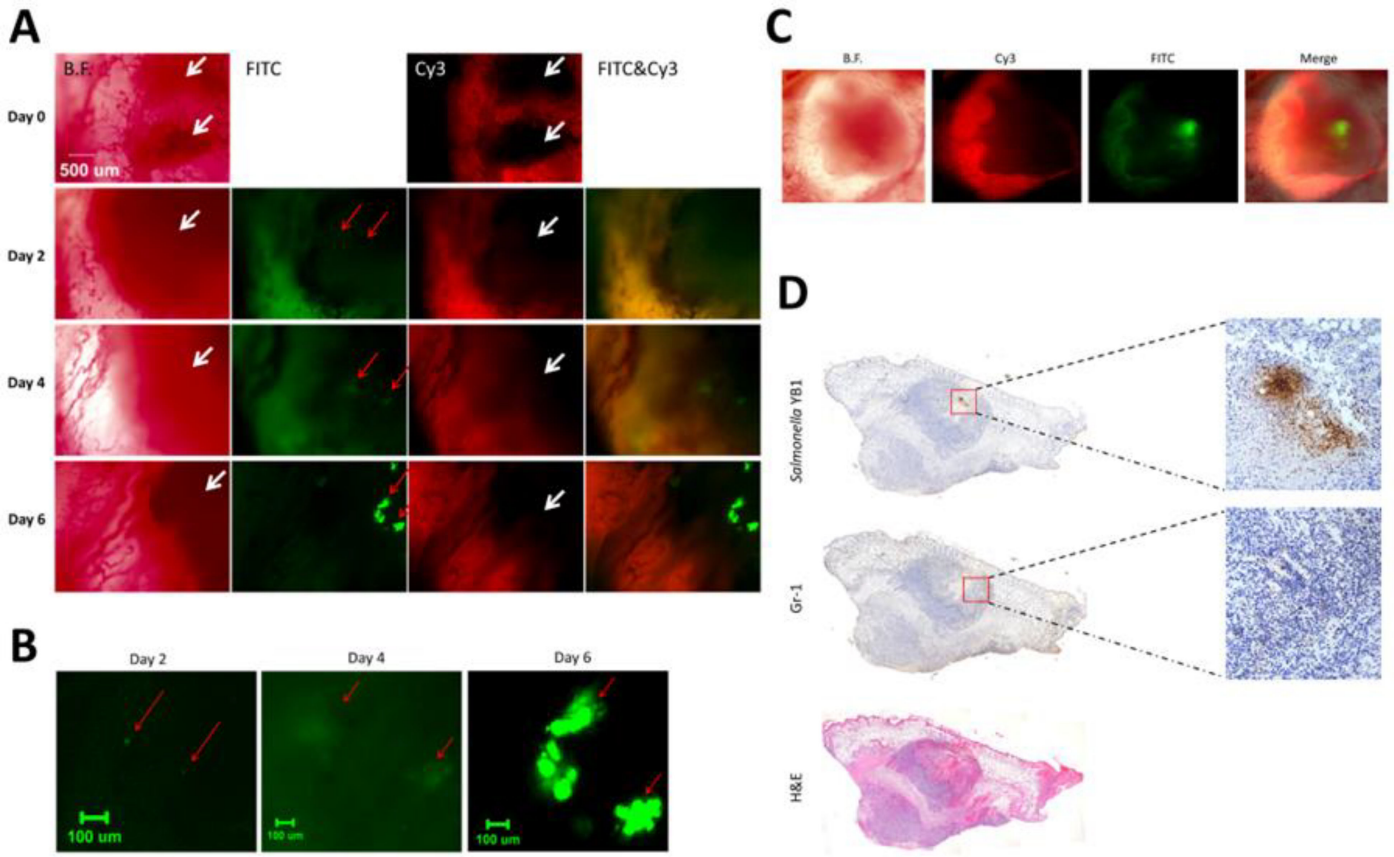
Late-phase dynamics of YB1-induced intratumoral colonization. **(A, B)** EGFP-labeled YB1 captured on days 2, 4, and 6. White arrow: hypoxic region; Red arrow: YB1. **(C)** Images captured on day 6. **(D)** H&E and immunohistochemistry staining for *Salmonella* and Gr-1 in YB1-treated tumors.

After 24 hours of YB1 treatment, the cytokine response in the systemic circulation subsided, leading to a more balanced state between the bacteria and host immune cells. To further elucidate these processes, we continuously monitored one MDA-MB-231 tumor-bearing mouse for 6 days (**Figure 7A**). On the second day post-administration, a small number of YB1 were identified in the thrombosed regions of the tumor. By the fourth day, these bacteria began to form small colonies, which significantly increased in size by the sixth day (**Figure 7B**). Continuous monitoring of another mouse revealed that as the bacterial colonies expanded, by day six they appeared to move away from the damaged vessels, colonizing primarily in the hypoxic areas of the tumor center and certain avascular regions (**Figure 7C**). At the study’s endpoint, the mice were euthanized with excessive CO_2_, and the tumors were excised for detailed histological analysis. The results confirmed the formation of YB1 colonies within the tumor, surrounded by recruited neutrophils (**Figure 7D**).

**Figure 8:**
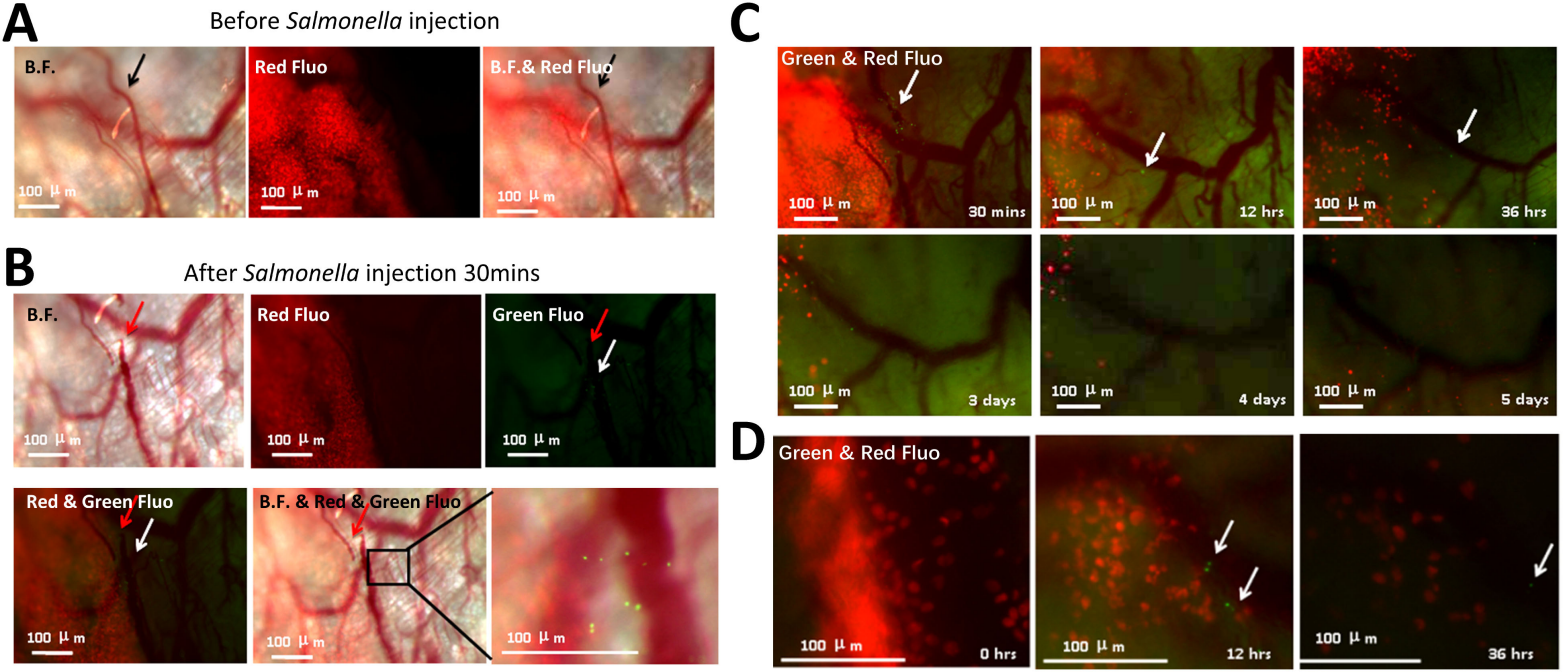
YB1 inhibitory effect on early-stage tumor. **(A)** Images of the tumor and associated blood vessels. The tumor-associated blood vessel (indicated by a black arrow) supplies nutrients and oxygen to the adjacent tumor. **(B)** After 30 minutes of YB1 treatment, YB1 disrupted the blood supply to the tumor (indicated by a red arrow). **(C)** Time-lapse tracking of tumor regression caused by YB1 treatment from 30 minutes to 5 days. **(D)** Apoptosis of cancer cells induced by YB1 after 12 and 36 hours. Green signal: YB1; Red signal: tdTomato-labeled MDA-MB-231 cancer cells. White arrows: YB1 distributions. Scale bars: 100 μm.

To evaluate the inhibitory effect of YB1 on early-stage tumors, we administered YB1 treatment 3 days after tumor implantation. At this point, the tumor was primarily supplied by two distinct blood vessels (**Figure 8A**). Notably, within just 30 minutes post-injection (**Figure 8B**), YB1-induced thrombosis led to vascular disruption. By 12 hours post-treatment, the tumor had already begun to regress significantly, despite the detection of a few YB1 within the occluded vessels. By day five, approximately 99% cancer cells had been eliminated (**Figure 8C**), and imaging data confirmed widespread apoptosis (**Figure 8D**), which is directly linked to the cutoff of oxygen supply. These results strongly indicate that YB1-induced thrombosis in tumor vessels is an effective strategy for eradicating early-stage tumors.

## 3 Discussion

Solid tumors are characterized by aberrant angiogenesis, leading to a disorganized and dysfunctional vascular network that is crucial for tumor invasion and metastasis (22, 23). Tumor-targeting bacteria, such as *Salmonella*, have garnered significant attention for their intrinsic ability to selectively colonize and proliferate within the tumor microenvironment (32–35). Some have already advanced to clinical stages. For instance, in a completed Phase I clinical trial (NCT00004988) (30), intravenous infusion of *Salmonella* VNP20009 at doses of 10^6^ to 10^9^ CFU/m² demonstrated a favorable safety profile and a certain capacity for tumor colonization. In another Phase II trial (NCT04589234) combining an attenuated *Salmonella* strain carrying the human interleukin-2 (IL-2) gene with standard chemotherapy (FOLFIRINOX or gemcitabine/nab-paclitaxel) (31), patients exhibited a trend towards improved median progression-free survival (mPFS) and median overall survival (mOS) compared to historical controls, with no serious adverse events attributed to the Salmonella-IL2 agent. However, despite the promise of bacterial cancer therapy, the precise mechanisms by which *Salmonella* interacts with the vasculature to inhibit tumor growth, particularly the exact spatiotemporal dynamics, remain to be fully elucidated.

In our previous work (16), we developed a *Salmonella* strain, YB1, by placing the essential *asd* gene under the control of a hypoxia-inducible promoter. This modification restricts YB1 survival to anaerobic conditions without compromising its other functions. Specifically, we have previously investigated the performance of YB1 in both immunocompetent BALB/c mice bearing CT26 colon carcinoma (17) and immunodeficient nude mice bearing MDA-MB-231 breast cancer (16). The results demonstrated that YB1 exhibits robust colonization and anti-tumor activity in both models, indicating that its targeting and colonization capabilities are effective regardless of the host’s immune status. Building on this foundation, the present study utilizes an intravital imaging system to track the biodistribution of YB1, tumor progression, and aberrant vascular dynamics in real-time in nude mice bearing MDA-MB-231 xenografts for the first time. Our findings reveal the significant therapeutic potential of the oncolytic YB1 in specifically targeting and disrupting tumor-associated vasculature.

Our *in vitro* and *in vivo* results strongly support the function of YB1 as a potent vascular disrupting agent. We demonstrated that YB1 directly damages vascular endothelial cells, induces their apoptosis, leading to the complete inhibition of tube formation. This pro-coagulant effect was further validated *in vivo*, where YB1 treatment significantly reduced tumor hemoglobin levels, indicating decreased vascular density and suppressed intratumoral blood supply.

Furthermore, our real-time intravital imaging enabled us to precisely characterize the dynamic interplay between YB1-mediated vascular disruption and bacterial colonization. Within 30 minutes of intravenous administration, a subset of bacteria was observed to specifically lodge in the “Shoulder Structure” of the rough, irregular lumens or the complex, tortuous “Maze Structure” of the tumor-associated vasculature. Repeated impacts on the nearby vascular endothelium caused endothelial cell damage and triggered intravascular thrombosis. For some tumor-associated vessels that did not exhibit immediate damage within 30 minutes, significant injury was typically observed within 24 hours post-treatment, concurrent with elevated TNF-α levels. As the coagulation cascade progressed, the tumor’s blood supply was occluded, creating a localized hypoxic environment that facilitated the efficient colonization and proliferation of YB1 within the tumor tissue. Ultimately, this synergy between vascular disruption and subsequent YB1 proliferation in the hypoxic tumor core led to extensive cancer cell apoptosis and significant tumor regression, particularly in early-stage tumors, even with a minimal bacterial presence within the damaged vessels. This aligns with the findings of Forbes et al. (24), who noted the difficulty of stable bacterial adhesion within blood vessels, and further clarifies the mechanism by which a small quantity of YB1 can efficiently induce tumor regression. This study not only supplements and deepens our understanding of YB1’s mechanism of action but also unveils the dynamic process by which YB1 actively remodels the tumor microenvironment to promote its own colonization, thereby advancing our comprehension of oncolytic bacterial therapy.

Drawing from previous literature, such as the work by Leschner et al., it is established that *Salmonella*-based therapy induces a broad spectrum of cytokines (e.g., IL-6, MCP-1, IFN-γ). Importantly, TNF-α is recognized as the key mediator driving tumor vascular hemorrhage and disruption. In this study, due to limitations of the mouse tumor model, the tumor sample volume available for ELISA analysis is extremely limited, which objectively restricts our ability to perform multiplex detection simultaneously. Consequently, we prioritized TNF-α as the primary analyte due to its direct mechanistic relevance to our observed vascular phenotype. The elevated TNF-α levels detected are interpreted as a direct outcome of host innate immune activation. Notably, no signs of severe toxicity (such as significant weight loss or mortality) were observed in the treated mice, suggesting that this transient immune activation did not precipitate a lethal cytokine storm and remained within a controllable, therapeutic window. It is crucial to recognize that a core mechanism of oncolytic bacterial therapy is to function as a potent immunoadjuvant. The initial innate immune response triggered by the bacteria is a prerequisite for breaking immune tolerance and initiating a subsequent adaptive, tumor-specific cellular immunity. While the present investigation focused on TNF-α, we acknowledge that comprehensive cytokine profiling will be essential in future studies to fully characterize the systemic immune response and further delineate the safety profile.

Although our intravital imaging system has provided profound insights, this study has limitations that warrant future investigation. Our findings suggest that while YB1 is highly effective against early-stage tumors via thrombosis in the tumor-associated vasculature, its efficacy in eradicating large, established tumors remains to be optimized. Future research should explore strategies to enhance the penetration and persistence of YB1 within large tumors. Potential approaches include genetic engineering to further improve its hypoxia-dependent proliferation or combinatorial strategies with immunotherapy or chemotherapy to achieve synergistic effects. Furthermore, YB1 could be engineered and express therapeutic transgenes aimed to enhance both safety profile and therapeutic efficacy. Moreover, for deep-seated large tumors such as hepatocellular or pancreatic carcinoma, in addition to conventional intratumoral injection and intravenous administration, transcatheter arterial infusion can be employed to deliver YB1 directly into the tumor-feeding arteries. Coupled with our vascular disruption mechanism, this strategy could not only significantly increase local bacterial concentration but also induce thrombosis, thereby blocking tumor blood supply and promoting tumor regression.

Additionally, regarding the molecular mechanism of vascular disruption, the precise upstream signaling pathways remain to be fully elucidated. We hypothesize that this phenomenon results from a synergistic effect of multiple factors: first, the aberrant hemodynamics of the tumor vasculature facilitate the physical entrapment of YB1, thereby increasing its residence time on the endothelial surface. Subsequently, this prolonged contact is thought to promote T3SS-mediated cellular invasion while simultaneously triggering a robust inflammatory response via the lipopolysaccharide (LPS)-TLR4 axis. The convergence of these events likely leads to endothelial apoptosis and vascular damage. In the future, utilizing various bacterial strains—such as T3SS-deficient *salmonella*, LPS-deficient *salmonella*, *Shigella* and *Escherichia coli* etc.—to elucidate whether these specific pathways drive vascular destruction will be a major focus of our work.

## 4 Materials and methods

### 4.1 Bacterial strains, animals, cell lines, and chemicals

*Salmonella* enterica strain YB1 was engineered as previously described and labeled with EGFP. Eight-week-old female nu/nu athymic mice were obtained from the Laboratory Animal Unit of the University of Hong Kong. The research protocols were approved by the Committee on the Use of Live Animals in Teaching and Research of the University of Hong Kong (CULATR 1685-08). The MDA-MB-231 breast cancer cell line was purchased from the American Type Culture Collection and labeled with nuclear tdTomato. HUVEC cells were obtained from Dr. E.H.C. Tang in the Department of Pharmacology & Pharmacy at HKU. Cells were routinely cultured under conditions specified by the manufacturer.

### 4.2 Apoptosis assay

Cells were harvested and stained using the Annexin-V/Propidium Iodide (PI) assay kit (BD Biosciences), followed by flow cytometric test (BD LSR Fortessa). BD LSR Fortessa Analyzer and FlowJo_v10.6.2 were used for flow cytometric analysis.

### 4.3 *In vivo* studies

Eight-week-old mice were used for these studies. Titanium window chambers were surgically implanted on the mice under anesthesia via intraperitoneal injection of ketamine (100 mg/kg) and xylazine (10 mg/kg). A 20 μl suspension (20,000 cells) of MDA-MB-231-tdTomato cells was injected into the dorsal skin fold, and a glass coverslip (diameter = 12 mm, No. 2, Erie Scientific, Portsmouth, New Hampshire) was placed over the exposed tissue. YB1 administration occurred on day 7 after tumor implantation.

### 4.4 ELISA assay

TNF-α levels were quantified using a mouse TNF-α ELISA kit (R&D Systems, Minneapolis, MN).

### 4.5 Histology and immunohistochemistry

Tumor samples were fixed immediately in 4% paraformaldehyde. After incubation, the samples were washed and dehydrated in graded ethanol. Following appropriate permeation in xylene, the fixed tissues were embedded in paraffin and cut into 7 μm sections. The sections were deparaffinized in xylene twice and rehydrated in descending concentrations of ethanol. Standard Hematoxylin–Eosin (H&E) staining of paraffin-embedded tissue was performed for histological examination (Anti-Salmonella antibody, abcam, ab35156; Anti-Gr-1 antibody, BD Biosciences, 560454).

### 4.6 Determination of hemoglobin content in tumors

Tumors were weighed, homogenized in PBS buffer, and centrifuged; the hemoglobin content in the supernatant was analyzed using Drabkin’s reagent (Sigma-Aldrich) and normalized to the weight.

### 4.7 Statistical analysis

All statistical analyses were performed using Prism software (GraphPad Prism). Statistical comparisons between two groups were evaluated using Student’s t-test. Differences were considered statistically significant when the *p* value was less than 0.05.

## 5 Conclusions

This study, using an intravital imaging system, for the first time elucidates in real-time and dynamically *in vivo* the distribution of the genetically engineered oncolytic bacterium YB1 and its effects on tumor vasculature. We have preliminarily defined the core mechanism of its tumor targeting and colonization. First, YB1 is retained in “ Shoulder Structure “ or “Maze Structure “ of tumor blood vessels, promoting direct interaction with vascular endothelial cells. This induces endothelial damage, apoptosis, thereby specifically triggering thrombosis in tumor vessels. This immediate, early-phase (within 30 minutes post-injection) vascular occlusion effectively cuts off the tumor’s blood, nutrient, and oxygen supply without affecting normal blood vessels. Second, the hypoxic tumor microenvironment resulting from vascular thrombosis creates a favorable condition for the hypoxia-activated engineered YB1 to migrate into, colonize, and proliferate within the tumor tissue. Ultimately, the synergy between this vascular disruption and the subsequent colonization and proliferation of bacteria within the hypoxic tumor leads to widespread cancer cell apoptosis and significantly promotes tumor regression, demonstrating exceptional efficacy, especially against early-stage tumors.

In conclusion, this research provides direct evidence of the spatiotemporal dynamics of YB1-mediated tumor vascular disruption and bacterial colonization, laying a solid foundation for future exploration and optimization of oncolytic bacteria for effective clinical cancer therapy.

## Data Availability

The original contributions presented in the study are included in the article/**Supplementary Material**. Further inquiries can be directed to the corresponding author.

## Supplementary material

Supplementary Figure 1 Shoulder structure.

Supplementary Video 1 A case of “ Shoulder Structure “ of tumor blood vessels.

Supplementary Video 2 Another case of “ Shoulder Structure “ of tumor blood vessels.

Supplementary Video 3 “Maze Structure “ of tumor blood vessels.

Supplementary Video 4 Within 30 minutes after systemic administration, fluorescence was detectable, showing no effects on normal blood vessels.

Supplementary Video 5 The third case of “ Shoulder Structure “ of tumor blood vessels.

Supplementary Video 6 Another case of “Maze Structure “ of tumor blood vessels.

## References

[1] Sung H Ferlay J Siegel RL Laversanne M Soerjomataram I Jemal A . Global cancer statistics 2020: GLOBOCAN estimates of incidence and mortality worldwide for 36 cancers in 185 countries. CA Cancer J Clin. (2021) 71:209–49. doi: 10.3322/caac.21660, PMID: 33538338

[2] Dagogo-Jack I Shaw AT . Tumour heterogeneity and resistance to cancer therapies. Nat Rev Clin Oncol. (2018) 15:81–94. doi: 10.1038/nrclinonc.2017.166, PMID: 29115304

[3] Sharma P Allison JP . The future of immune checkpoint therapy. Science. (2015) 348:56–61. doi: 10.1126/science.aaa8172, PMID: 25838373

[4] Forbes NS . Engineering the perfect (bacterial) cancer therapy. Nat Rev Cancer. (2010) 10:785–94. doi: 10.1038/nrc2934, PMID: 20944664 PMC3756932

[5] Gupta KH Nowicki C Giurini EF Marzo AL Zloza A . Bacterial-based cancer therapy (BBCT): recent advances, current challenges, and future prospects for cancer immunotherapy. Vaccines (Basel). (2021) 9:1497. doi: 10.3390/vaccines9121497, PMID: 34960243 PMC8707929

[6] Liang S Wang C Shao Y Wang Y Xing D Geng Z . Recent advances in bacteria-mediated cancer therapy. Front Bioeng. Biotechnol. (2022) 10:1026248. doi: 10.3389/fbioe.2022.1026248, PMID: 36312554 PMC9597243

[7] Feng X He P Zeng C Li YH Das SK Li B . Novel insights into the role of Clostridium novyi-NT related combination bacteriolytic therapy in solid tumors. Oncol Lett. (2021) 21:110. doi: 10.3892/ol.2020.12371, PMID: 33376543 PMC7751347

[8] McCarthy EF . The toxins of William B. Coley and the treatment of bone and soft-tissue sarcomas. Iowa. Orthop. J. (2006) 26:154–8., PMID: 16789469 PMC1888599

[9] Le DT Wang-Gillam A Picozzi V Greten TF Crocenzi T Springett G . Safety and survival with GVAX pancreas prime and Listeria Monocytogenes-expressing mesothelin (CRS-207) boost vaccines for metastatic pancreatic cancer. J Clin Oncol. (2015) 33:1325–33. doi: 10.1200/JCO.2014.57.4244, PMID: 25584002 PMC4397277

[10] Kamble NS Thomas S Madaan T Ehsani N Sange S Tucker K . Engineered bacteria as an orally administered anti-viral treatment and immunization system. Gut. Microbes. (2025) 17:2500056. doi: 10.1080/19490976.2025.2500056, PMID: 40340796 PMC12064065

[11] Canale FP Basso C Antonini G Perotti M Li N Sokolovska A . Metabolic modulation of tumours with engineered bacteria for immunotherapy. Nature. (2021) 598:662–6. doi: 10.1038/s41586-021-04003-2, PMID: 34616044

[12] Chowdhury S Castro S Coker C Hinchliffe TE Arpaia N Danino T . Programmable bacteria induce durable tumor regression and systemic antitumor immunity. Nat Med. (2019) 25:1057–63. doi: 10.1038/s41591-019-0498-z, PMID: 31270504 PMC6688650

[13] Low KB Ittensohn M Le T Platt J Sodi S Amoss M . Lipid A mutant Salmonella with suppressed virulence and TNFalpha induction retain tumor-targeting in *vivo*. Nat Biotechnol. (1999) 17:37–41. doi: 10.1038/5205, PMID: 9920266

[14] Clairmont C Lee KC Pike J Ittensohn M Low KB Pawelek J . Biodistribution and genetic stability of the novel antitumor agent VNP20009, a genetically modified strain of Salmonella typhimurium. J Infect Dis. (2000) 181:1996–2002. doi: 10.1086/315497, PMID: 10837181

[15] Hoffman RM . Tumor-targeting salmonella typhimurium A1-R: an overview. Methods Mol Biol. (2016) 1409:1–8. doi: 10.1007/978-1-4939-3515-4, PMID: 26846797

[16] Yu B Yang M Shi L Yao Y Jiang Q Li X . Explicit hypoxia targeting with tumor suppression by creating an "obligate" anaerobic Salmonella Typhimurium strain. Sci Rep. (2012) 2:436. doi: 10.1038/srep00436, PMID: 22666539 PMC3365283

[17] Yu B Shi L Zhang BZ Zhang KE Peng X Niu HB . Obligate anaerobic Salmonella typhimurium strain YB1 treatment on xenograft tumor in immunocompetent mouse model. Oncol Lett. (2015) 10:1069–74. doi: 10.3892/ol.2015.3302, PMID: 26622627 PMC4509430

[18] Lin Q Rong L Jia X Li R Yu B Hu J . IFN-γ-dependent NK cell activation is essential to metastasis suppression by engineered Salmonella. Nat Commun. (2021) 12:2537. doi: 10.1038/s41467-021-22755-3, PMID: 33953170 PMC8099885

[19] Ning BT Yu B Chan S Chan JL Huang JD Chan GC . Treatment of neuroblastoma with an engineered "Obligate" Anaerobic salmonella typhimurium strain YB1. J Cancer. (2017) 8:1609–18. doi: 10.7150/jca.18776, PMID: 28775780 PMC5535716

[20] Li CX Yu B Shi L Geng W Lin QB Ling CC . 'Obligate' anaerobic Salmonella strain YB1 suppresses liver tumor growth and metastasis in nude mice. Oncol Lett. (2017) 13:177–83. doi: 10.3892/ol.2016.5453, PMID: 28123538 PMC5245073

[21] Forster JC Harriss-Phillips WM Douglass MJ Bezak E . A review of the development of tumor vasculature and its effects on the tumor microenvironment. Hypoxia (Auckl). (2017) 5:21–32. doi: 10.2147/HP.S133231, PMID: 28443291 PMC5395278

[22] Farnsworth RH Lackmann M Achen MG Stacker SA . Vascular remodeling in cancer. Oncogene. (2014) 33:3496–505. doi: 10.1038/onc.2013.304, PMID: 23912450

[23] Ganai S Arenas RB Sauer JP Bentley B Forbes NS . In tumors Salmonella migrate away from vasculature toward the transition zone and induce apoptosis. Cancer Gene Ther. (2011) 18:457–66. doi: 10.1038/cgt.2011.10, PMID: 21436868 PMC3117926

[24] Jain RK Munn LL Fukumura D . Dissecting tumour pathophysiology using intravital microscopy. Nat Rev Cancer. (2002) 2:266–76. doi: 10.1038/nrc778, PMID: 12001988

[25] Forbes NS Munn LL Fukumura D Jain RK . Sparse initial entrapment of systemically injected Salmonella typhimurium leads to heterogeneous accumulation within tumors. Cancer Res. (2003) 63:5188–93., PMID: 14500342

[26] Leschner S Westphal K Dietrich N Viegas N Jablonska J Lyszkiewicz M . Tumor invasion of Salmonella enterica serovar Typhimurium is accompanied by strong hemorrhage promoted by TNF-alpha. PloS One. (2009) 4:e6692. doi: 10.1371/journal.pone.0006692, PMID: 19693266 PMC2724709

[27] Alieva M Ritsma L Giedt RJ Weissleder R van Rheenen J . Imaging windows for long-term intravital imaging: General overview and technical insights. Intravital. (2014) 3:e29917. doi: 10.4161/intv.29917, PMID: 28243510 PMC5312719

[28] Rajabi M Mousa SA . The role of angiogenesis in cancer treatment. Biomedicines. (2017) 5:34. doi: 10.3390/biomedicines5020034, PMID: 28635679 PMC5489820

[29] Hashizume H Baluk P Morikawa S McLean JW Thurston G Roberge S . Openings between defective endothelial cells explain tumor vessel leakiness. Am J Pathol. (2000) 156:1363–80. doi: 10.1016/S0002-9440(10)65006-7, PMID: 10751361 PMC1876882

[30] Toso JF Gill VJ Hwu P Marincola FM Restifo NP Schwartzentruber DJ . Phase I study of the intravenous administration of attenuated Salmonella typhimurium to patients with metastatic melanoma. J Clin Oncol. (2002) 20:142–52. doi: 10.1200/JCO.2002.20.1.142, PMID: 11773163 PMC2064865

[31] Saltzman D Kavan P Augustin L Schottel J Lee JT Moradian J . Influence of salmonella-IL2 in combination with FOLFIRINOX on overall and progression-free survival in stage IV metastatic pancreatic cancer. J Clin Oncol. (2025) 43:2571–1. doi: 10.1200/JCO.2025.43.16_suppl.2571

[32] Aganja RP Sivasankar C Senevirathne A Lee JH . Salmonella as a promising curative tool against cancer. Pharmaceutics. (2022) 14:2100. doi: 10.3390/pharmaceutics14102100, PMID: 36297535 PMC9609134

[33] Liang K Liu Q Li P Luo H Wang H Kong Q . Genetically engineered Salmonella Typhimurium: Recent advances in cancer therapy. Cancer Lett. (2019) 448:168–81. doi: 10.1016/j.canlet.2019.01.037, PMID: 30753837

[34] Guo Y Chen Y Liu X Min JJ Tan W Zheng JH . Targeted cancer immunotherapy with genetically engineered oncolytic Salmonella typhimurium. Cancer Lett. (2020) 469:102–10. doi: 10.1016/j.canlet.2019.10.033, PMID: 31666180

[35] Chen W Zhu Y Zhang Z Sun X . Advances in Salmonella Typhimurium-based drug delivery system for cancer therapy. Adv Drug Delivery Rev. (2022) 185:114295. doi: 10.1016/j.addr.2022.114295, PMID: 35429576

