# Supplementary figures and images for: Intravital imaging reveals spatiotemporal dynamics of oncolytic Salmonella YB1-induced intratumoral vascular thrombosis and tumor targeting
Source: Front Immunol. 2026 Jan 16;16:1733164. doi: 10.3389/fimmu.2025.1733164 (PMC12855406; doi:10.3389/fimmu.2025.1733164)

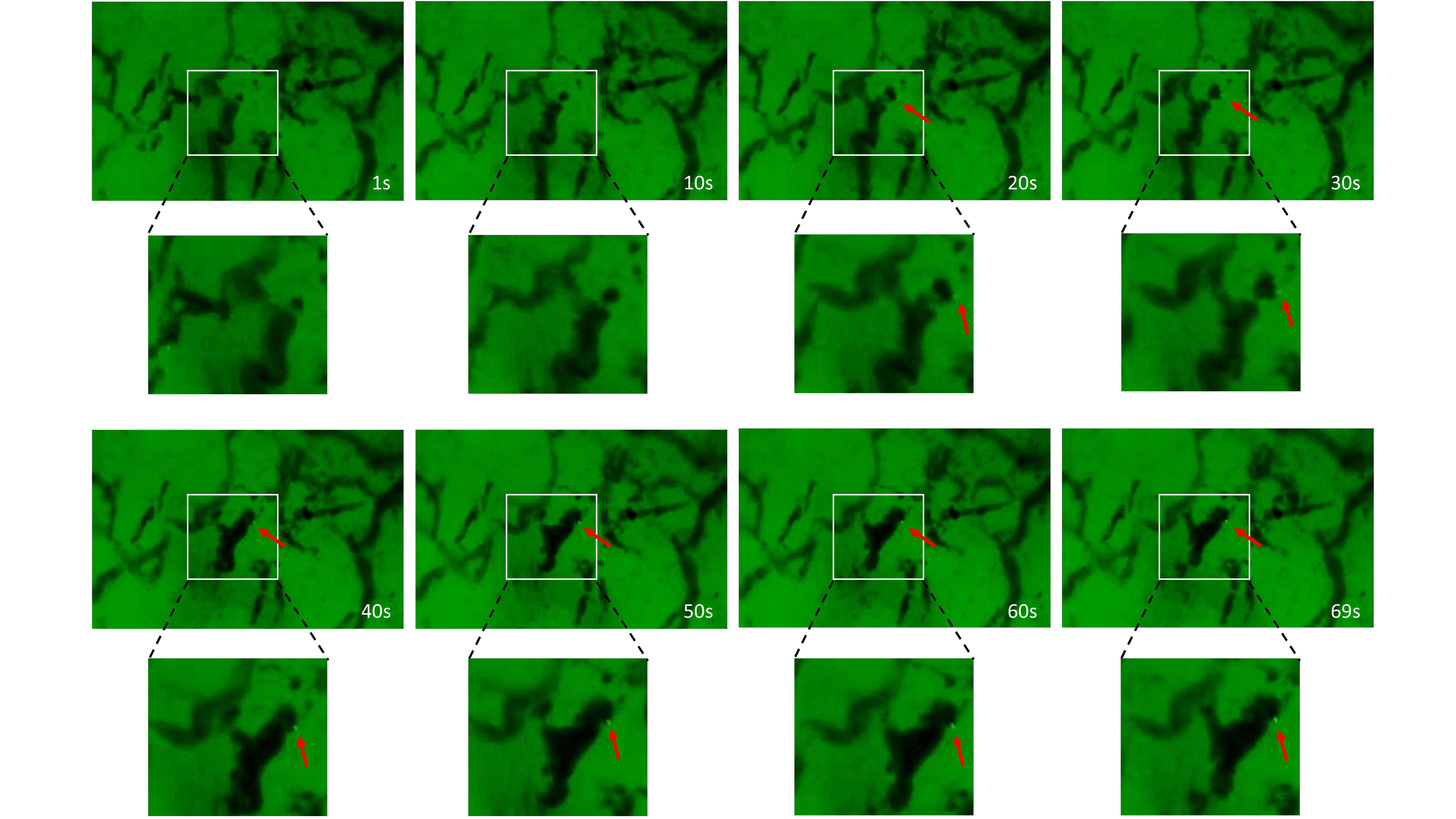

Supplement: Supplementary Figure 1 — Shoulder structure. [file Image1.tif]
